# Supplementary material for: Pediatric Respiratory Hospitalizations in the Pre-COVID-19 Era: The Contribution of Viral Pathogens and Comorbidities to Clinical Outcomes, Valencia, Spain
Source: Viruses. 2024 Sep 25;16(10):1519. doi: 10.3390/v16101519 (PMC11512402; doi:10.3390/v16101519)
Supplement: Supplementary file 1 [file viruses-16-01519-s001.zip › viruses-3197723-supplementary.pdf]

SUPPLEMENTARY DATA/INFORMATION

Viruses, special issue « Influenza and Other Respiratory Viruses: Prevention, Diagnosis, Treatment"

Pediatric respiratory hospitalizations in the pre-COVID-19 era: The contribution of viral pathogens and comorbidities to clinical outcomes, Valencia, Spain

Valérie Bosch Castells[1], Ainara Mira-Iglesias[2,3], F. Xavier López-Labrador[3,4], Beatriz Mengual-Chuliá[3,4], Mario Carballido-Fernández[5,6], Miguel Tortajada-Girbés[7], Joan Mollar-Maseres[8], Joan Puig-Barberà[2], Javier Díez-Domingo[2,3], Sandra S. Chaves[1]

[1]*Modelling, Epidemiology and Data Science Department, Sanofi Vaccines, Lyon, France*

[2]*Área de Investigación en Vacunas. Fundación para el Fomento de la Investigación Sanitaria y Biomédica de la Comunitat Valenciana (FISABIO-Public Health). Valencia, Spain*

[3] *Consorcio de Investigación Biomédica de Epidemiología y Salud Pública (CIBERESP), Instituto de Salud Carlos III, Madrid, Spain*

[4] *Área de Genómica y Salud. Fundación para el Fomento de la Investigación Sanitaria y Biomédica de la Comunitat Valenciana (FISABIO-Public Health). Valencia, Spain .*

[5]*Hospital General Universitario de Castellón, Castellón, Spain.*

[6]*Departamento de Medicina, Universidad CEU Cardenal Herrera, Castellón, Spain.*

[7]*Hospital Universitario Doctor Peset, Valencia, Spain.*

[8]*Hospital Universitario y Politécnico La Fe, Valencia, Spain.*

Corresponding author:

Valérie Bosch Castells, Sanofi, 14 Espace Henry Vallée, 69007 Lyon, France

Tel: +33 788 258 910; E-mail: valerie.boschcastells@sanofi.com

Table S1. International Classification of Diseases (ICD) codes used to classify complications

Group/type	Description	ICD-9	ICD-10	n
**Sinorespiratory**				
Bronchitis/Bronchiolitis	Acute bronchiolitis	466.1	J21	238
	Acute bronchiolitis due to human metapneumovirus	-	J21.1	2
	Acute bronchiolitis due to other infectious organisms	466.19	-	589
	Acute bronchiolitis due to other specified organisms	-	J21.8	4
	Acute bronchiolitis due to respiratory syncytial virus	-	J21.0	154
	Acute bronchiolitis due to respiratory syncytial virus (RSV)	466.11	-	777
	Acute bronchiolitis, unspecified	-	J21.9	85
	Acute bronchitis	466.0	J20	216
	Acute bronchitis and bronchiolitis	466	-	274
	Acute bronchitis due to other specified organisms	-	J20.8	1
	Acute bronchitis due to respiratory syncytial virus	-	J20.5	10
	Acute bronchitis due to streptococcus	-	J20.2	1
	Acute bronchitis, unspecified	-	J20.9	53
	Bronchitis, not specified as acute or chronic	490	J40	90
Asthma exacerbation/Reactive airways	Acute bronchospasm	519.11	-	416
	Asthma, unspecified type, with (acute) exacerbation	493.92	-	19
	Asthma, unspecified type, with status asthmaticus	493.91	-	9
	Cough variant asthma	493.82	-	1
	Exercise induced bronchospasm	493.81	-	1
	Extrinsic asthma with (acute) exacerbation	493.02	-	17
	Extrinsic asthma with status asthmaticus	493.01	-	1
	Intrinsic asthma with (acute) exacerbation	493.12	-	1
	Status asthmaticus	-	J46	12
Laryngitis/Pharyngitis	Acute laryngitis	464.0	J04.0	12
	Acute laryngitis and tracheitis	464	-	7
	Acute laryngitis with obstruction	464.01	-	2
	Acute laryngitis without mention of obstruction	464.00	-	17
	Acute laryngopharyngitis	465.0	-	2
	Acute laryngotracheitis	464.2	J04.2	7
	Acute nasopharyngitis [common cold]	460	J00	39
	Acute pharyngitis	462	J02	16
	Acute pharyngitis, unspecified	-	J02.9	1
	Acute tonsillitis	463	J03	54
	Acute tonsillitis, unspecified	-	J03.9	5
	Enteroviral vesicular pharyngitis	-	B08.5	1
	Herpes viral gingivostomatitis and pharyngotonsillitis	-	B00.2	3

	Scarlet fever	-	A38	6
	Streptococcal pharyngitis	-	J02.0	1
	Streptococcal sore throat	034.0	-	7
	Streptococcal sore throat and scarlet fever	34, 034	-	1
Mastoiditis	Acute mastoiditis with other complications	383.02	-	1
	Mastoiditis, unspecified	-	H70.9	1
Otitis media	Acute allergic serous otitis media	381.4	-	1
	Acute mucoid otitis media	381.02	-	1
	Acute nonsuppurative otitis media	381.0	-	1
	Acute nonsuppurative otitis media, unspecified	381.00	-	5
	Acute serous otitis media	381.01	-	1
	Acute suppurative otitis media	382.0	-	2
	Nonsuppurative otitis media	-	H65	8
	Nonsuppurative otitis media and eustachian tube disorders	381	-	3
	Nonsuppurative otitis media, unspecified	-	H65.9	1
	Otitis media in diseases classified elsewhere	-	H67	2
	Otitis media, unspecified	-	H66.9	23
	Suppurative and unspecified otitis media	382	-	2
	Suppurative otitis media, unspecified	-	H66.4	1
	Unspecified otitis media	382.9	-	90
	Unspecified suppurative otitis media	382.4	-	1
Respiratory failure	Acute and chronic respiratory failure	518.84	-	2
	Acute respiratory failure	518.81	J96.0	16
	Apnea	786.03	-	3
	Other pulmonary insufficiency, not elsewhere classified	518.82	-	6
	Respiratory arrest	-	R09.2	2
	Respiratory failure, not elsewhere classified	-	J96	1
Sinusitis	Acute ethmoidal sinusitis	461.2	-	1
	Acute sinusitis, unspecified	461.9	J01.9	2
Tuberculosis	Other specified pulmonary tuberculosis	011.8	-	2
	Pulmonary tuberculosis, unspecified, tubercle bacilli not found (in sputum) by microscopy, but found by bacterial culture	011.94	-	1
	Pulmonary tuberculosis, unspecified, unspecified	011.90	-	1
	Unspecified pulmonary tuberculosis	011.9	-	2
Pneumothorax	Other pneumothorax and air leak	512.8	-	1
	Pulmonary collapse	518.0	J98.1	102
Pneumonia	Abscess of lung with pneumonia	-	J85.1	1
	Bacterial pneumonia, unspecified	482.9	-	17
	Bronchopneumonia, organism unspecified	485	-	172
	Bronchopneumonia, unspecified	-	J18.0	27
	Chlamydial pneumonia	-	J16.0, J16	3
	Influenza due to identified 2009 H1N1 influenza virus with pneumonia	488.11	-	10
	Influenza due to identified avian influenza virus with pneumonia	488.01	-	2
	Influenza due to identified novel influenza A virus with pneumonia	488.81	-	5
	Influenza with pneumonia	487.0	-	2
	Influenza with pneumonia, seasonal influenza virus identified	-	J10.0	4
	Influenza with pneumonia, virus not identified	-	J11.0	9
	Lobar pneumonia, unspecified	-	J18.1	1
	Other bacterial pneumonia	482	-	4
	Parainfluenza virus pneumonia	-	J12.2	2
	Pneumococcal pneumonia [Streptococcus pneumoniae pneumonia]	481	-	34
	Pneumonia due to Hemophilus influenzae [H. influenzae]	482.2	-	1

	Pneumonia due to mycoplasma pneumoniae	483.0,	J15.7	6
	Pneumonia due to other specified bacteria	482.89	-	1
	Pneumonia due to other specified organism	483, 483.8	-	11
	Pneumonia due to other virus not elsewhere classified	480.8	-	6
	Pneumonia due to respiratory syncytial virus	480.1	-	10
	Pneumonia due to Streptococcus pneumoniae	-	J13	3
	Pneumonia, organism unspecified	486	J18	594
	Pneumonia, unspecified	-	J18.9	190
	Respiratory syncytial virus pneumonia	-	J12.1	21
	Unspecified acute lower respiratory infection	-	J22	12
	Viral pneumonia	480	-	4
	Viral pneumonia, not elsewhere classified	-	J12	2
	Viral pneumonia, unspecified	480.9	J12.9	24
Pleural effusion	Empyema without mention of fistula	510.9	-	2
	Other specified forms of effusion, except tuberculous	511.89	-	3
	Pleural effusion in conditions classified elsewhere	-	J91	1
	Pleural effusion, not elsewhere classified	-	J90	16
	Pleurisy with effusion, with mention of a bacterial cause other than tuberculosis	511.1	-	2
	Pleurisy without mention of effusion or current tuberculosis	511.0	-	1
	Unspecified pleural effusion	511.9	-	27
**Neurologic disorders**				
Encephalitis	Unspecified causes of encephalitis, myelitis, and encephalomyelitis	323.9	-	1
	Unspecified viral encephalitis	-	A86	1
Encephalopathy	Hypoxic-ischemic encephalopathy, unspecified	768.70	-	1
	Other and unspecified encephalopathy	-	G93.4	2
	Other encephalopathy	348.39	-	2
Nonfebrile seizures	Convulsions	780.3	-	11
	Convulsions, not elsewhere classified	-	R56	10
	Epilepsia partialis continua	345.7	-	1
	Epilepsia partialis continua, without mention of intractable epilepsy	345.70	-	1
	Epilepsy and recurrent seizures	345	G40	6
	Epilepsy unspecified	345.9	-	16
	Epilepsy, unspecified	-	G40.9	5
	Epilepsy, unspecified, without mention of intractable epilepsy	345.90	-	6
	Generalized convulsive epilepsy	345.1	-	1
	Generalized convulsive epilepsy, without mention of intractable epilepsy	345.10	-	1
	Generalized idiopathic epilepsy and epileptic syndromes	-	G40.3	6
	Localization-related (focal) (partial) epilepsy and epileptic syndromes with complex partial seizures	345.4	-	7
	Localization-related (focal) (partial) epilepsy and epileptic syndromes with complex partial seizures, with intractable epilepsy	345.41	-	1
	Localization-related (focal) (partial) epilepsy and epileptic syndromes with complex partial seizures, without mention of intractable epilepsy	345.40	-	2
	Localization-related (focal) (partial) epilepsy and epileptic syndromes with simple partial seizures	345.5	-	2

	Localization-related (focal) (partial) epilepsy and epileptic syndromes with simple partial seizures, without mention of intractable epilepsy	345.50	-	1
	Localization-related (focal) (partial) idiopathic epilepsy and epileptic syndromes with seizures of localized onset	-	G40.0	1
	Localization-related (focal)(partial) symptomatic epilepsy and epileptic syndromes with simple partial seizures	-	G40.1	1
	Other and unspecified convulsions	-	R56.8	5
	Other convulsions	780.39	-	51
	Other generalized epilepsy and epileptic syndromes	-	G40.4	3
Febrile seizures	Complex febrile convulsions	780.32	-	35
	Febrile convulsions	-	R56.0	64
	Febrile convulsions (simple), unspecified	780.31	-	252
	Status epilepticus	-	G41	1
Meningitis	Enteroviral meningitis	-	A87.0	1
	Meningitis due to enterovirus	47, 047	-	1
	Meningitis due to other organism	321	-	2
	Meningitis due to unspecified bacterium	320.9	-	1
	Meningitis, unspecified	322.9	G03.9	4
	Meningococcal meningitis	036.0	-	1
	Nonpyogenic meningitis	-	G03.0	1
	Pneumococcal meningitis	320.1	-	1
	Unspecified viral meningitis	047.9	-	13
**Metabolic disorders**				
Fluid and Electrolyte disorders	Acetonuria	791.6	-	3
	Acidosis	276.2	E87.2	25
	Alkalosis	276.3	-	2
	Dehydration	276.51	-	26
	Hypercalcemia	275.42	-	1
	Hyperosmolality and hypernatremia	-	E87.0	1
	Hyperosmolality and/or hypernatremia	276.0	-	1
	Hypo-osmolality and hyponatremia	-	E87.1	2
	Hypopotassemia	276.8	-	1
	Hypo-osmolality and/or hyponatremia	276.1	-	2
	Other transitory neonatal electrolyte disturbances	775.5	-	1
Diabetes	Diabetes with unspecified complication, type I [juvenile type], not stated as uncontrolled	250.91	-	1
	Diabetes with ketoacidosis	250.1	-	1
	Diabetes with ketoacidosis, type I [juvenile type], not stated as uncontrolled	250.11	-	1
	Type 1 diabetes mellitus with ketoacidosis	-	E10.1	3
	Type 1 diabetes mellitus, with unspecified complications	-	E10.8	2
**Hematology**				
Anemia	Aplastic anemia, unspecified	284.9	-	1
	Iron deficiency anemia, unspecified	280.9	D50.9	42
Neutropenia	Agranulocytosis	-	D70	3
	Drug induced neutropenia	288.03	-	1
	Neutropenia due to infection	288.04	-	4
	Neutropenia, unspecified	288.00	-	5
**Urology**				
Hydronephrosis	Hydronephrosis	591	-	6
Kidney	Acute kidney failure, unspecified	584.9	-	1
	Cystic kidney disease	753.1	-	1
	Nephrotic syndrome with unspecified pathological lesion in kidney	581.9	-	1
Nephritis	Acute pyelonephritis	590.1	-	11

	Acute pyelonephritis without lesion of renal medullary necrosis	590.10	-	7
	Acute tubulo-interstitial nephritis	-	N10	3
	Pyelonephritis, unspecified	590.80	-	4
	Unspecified nephritic syndrome, unspecified	-	N05.9	1
Urinary tract infection	Urinary tract infection of newborn	771.82	-	3
	Urinary tract infection, site not specified	599.0	N39.0	42
**Other**				
Candidiasis	Candidiasis	112	B37	9
	Candidal stomatitis	-	B37.0	1
	Candidiasis of mouth	112.0	-	15
	Candidiasis of other urogenital sites	112.2	-	1
	Candidiasis of skin and nails	112.3	-	4
	Candidiasis of unspecified site	112.9	-	3
Cardiovascular	Acute myocardial infarction of anterolateral wall, initial episode of care	410.01	-	1
	Angina pectoris	-	I20	1
	Atrial fibrillation	427.31	-	1
	Cerebral artery occlusion, unspecified with cerebral infarction	434.91	-	1
	Heart failure	428	-	1
	Heart failure, unspecified	428.9	I50.9	2
	Left heart failure	428.1	-	1
	Other specified cardiac dysrhythmias	427.89	-	1
	Pericarditis in bacterial diseases classified elsewhere	-	I32.0	1
	Subsequent myocardial infarction of anterior wall	-	I22	1
Conjunctivitis	Acute atopic conjunctivitis	372.05	-	1
	Acute conjunctivitis, unspecified	372.00	-	30
	Conjunctivitis	-	H10	5
	Conjunctivitis, unspecified	372.30	-	31
	Disorders of conjunctiva	372	-	11
	Neonatal conjunctivitis and dacryocystitis	771.6	-	7
	Other mucopurulent conjunctivitis	372.03	-	11
	Unspecified conjunctivitis	-	H10.9	5
Diarrhea/Colitis	Allergic gastroenteritis and colitis	558.3	-	1
	Campylobacter enteritis	-	A04.5	1
	Colitis, enteritis, and gastroenteritis of presumed infectious origin	009.1	-	24
	Diarrhea	787.91	-	11
	Diarrhea of presumed infectious origin	009.3	-	1
	Enteritis due to adenovirus	008.62	-	3
	Enteritis due to astrovirus	008.66	-	1
	Enteritis due to rotavirus	008.61	-	61
	Infectious colitis, enteritis, and gastroenteritis	009.0	-	49
	Infectious gastroenteritis and colitis, unspecified	-	A09	2
	Intestinal infection due to campylobacter	008.43	-	4
	Intestinal infection due to other organism, not elsewhere classified	008.8	-	3
	Intestinal infection due to yersinia enterocolitica	008.44	-	1
	Noninfective gastroenteritis and colitis, unspecified	-	K52.9	14
	Other and unspecified noninfectious gastroenteritis and colitis	558, 558.9	-	62
	Rotaviral enteritis	-	A08.0	5
	Salmonella enteritis	-	A02.0	3
	Salmonella gastroenteritis	003.0	-	8
Gastrointestinal bleeding	Acute haemorrhagic gastritis	-	K29.0	1
	Gastrointestinal hemorrhage	578	-	1
	Hematemesis	578.0	-	1

Hypoxemia	Hypoxemia	799.02	-	46
Kawasaki	Acute febrile mucocutaneous lymph node syndrome [MCLS]	446.1	-	5
Musculoskeletal	Infective myositis	728.0	M60.0	1
	Myopathy, unspecified	359.9	-	2
	Other specified anomalies of muscle, tendon, fascia, and connective tissue	756.89	-	1
	Other specified disorders of muscle	-	M62.8	1
	Unspecified disorder of muscle, ligament, and fascia	728.9	-	2
Sepsis	Bacteremia	790.7	-	29
	Meningococcemia	036.2	-	1
	Other septicemia due to gram-negative organisms	038.49	-	1
	Pneumococcal septicemia [Streptococcus pneumoniae septicemia]	038.2	-	2
	Sepsis	995.91	-	13
	Sepsis due to other Gram-negative organisms	-	A41.5	1
	Septic shock	785.52	-	2
	Sepsis, unspecified	-	A41.9	1
	Septicemia due to gram-negative organism, unspecified	038.40	-	1
	Septicemia [sepsis] of newborn	771.81	-	1
	Severe sepsis	995.92	-	1
	Other specified sepsis	-	A41.8	1
	Unspecified septicemia	038.9	-	6
Thrombocytopenia	Defibrination syndrome	286.6	-	1
	Essential thrombocythemia	238.71	-	3
	Secondary thrombocytopenia	287.4	D69.5	3
	Thrombocytopenia, unspecified	287.5	-	2
	Other secondary thrombocytopenia	287.49	-	4
Viremia	Viremia, unspecified	790.8	-	28

Table S2. International Classification of Diseases (ICD) codes by comorbidity

Comorbidity	ICD code description	ICD-9	ICD-10	n
Anemia	Anemia, unspecified	285.9	D64.9	63
	Congenital anemia	776.5	-	1
	Sickle-cell disease	282.6	-	1
	Sickle-cell disorders	-	D57	1
	Sickle-cell trait	-	D57.3	1
	Thalassemia minor	-	D56.3	1
Asthma	Asthma	493	J45, J45.1	38
	Asthma, unspecified	493.9	J45.9	18
	Asthma, unspecified type, unspecified	493.90	-	5
	Extrinsic asthma	493.0	-	28
	Extrinsic asthma, unspecified	493.00	-	2
	Intrinsic asthma	493.1	-	2
	Predominantly allergic asthma	-	J45.0	18
Autoimmune	Other demyelinating diseases of central nervous system	341.8	-	1
Diabetes	Diabetes mellitus without mention of complication, type I [juvenile type], not stated as uncontrolled	250.01	-	1
	Diabetes mellitus without mention of complication, type II or unspecified type, not stated as uncontrolled	250.00	-	2
	Diabetes mellitus without mention of complication	250.0	-	3
	Type 1 diabetes mellitus	-	E10	2
	Type 1 diabetes mellitus without complications	-	E10.9	1

	Unspecified diabetes mellitus	-	E14	1
Endocrine system disease other than diabetes	Congenital hypothyroidism	243	-	1
Heart disease	Aortic valve disorders	424.1	-	1
	Atrial septal defect	-	Q21.1	1
	Chronic pulmonary heart disease, unspecified	416.9	-	1
	Congestive heart failure, unspecified	428.0	-	2
	Heart disease, unspecified	429.9	I51.9	4
	Long QT syndrome	426.82	-	1
	Mitral valve disorders	424.0	-	1
	Patent ductus arteriosus	747.0	-	2
	Ostium secundum type atrial septal defect	745.5	-	6
	Other diseases of endocardium	424	-	1
	Other premature beats	427.69	-	1
	Other primary cardiomyopathies	425.4	-	2
	Other specified diseases of pericardium	423.8	-	1
	Right bundle branch block	426.4	-	2
	Sinoatrial node dysfunction	427.81	-	1
	Tetralogy of Fallot	745.2	-	2
	Unspecified congenital anomaly of heart	746.9	-	1
	Unspecified defect of septal closure	745.9	-	1
	Unspecified disease of pericardium	423.9	-	1
	Ventricular septal defect	745.4	-	7
Liver disease	Cirrhosis of liver without mention of alcohol	571.5	-	2
	Other chronic nonalcoholic liver disease	571.8	-	1
Lung disease	Agenesis, hypoplasia, and dysplasia of lung	748.5	-	2
	Bronchopulmonary dysplasia originating in the perinatal period	-	P27.1	2
	Chronic bronchitis	491	-	2
	Chronic obstructive pulmonary disease with (acute) exacerbation	-	J44.1	1
	Chronic respiratory disease arising in the perinatal period	770.7	-	4
	Congenital cystic lung	-	Q33.0	1
	Cystic fibrosis	277.0	E84	2
	Cystic fibrosis with pulmonary manifestations	-	E84.0	1
	Cystic fibrosis without mention of meconium ileus	277.00	-	1
	Emphysema	492	-	1
	Obstructive chronic bronchitis	491.2	-	3
	Obstructive chronic bronchitis with acute bronchitis	491.22	-	1
	Obstructive chronic bronchitis without exacerbation	491.20	-	10
	Other chronic pulmonary heart diseases	416.8	-	1
	Other diseases of lung	518	-	19
	Other diseases of lung, not elsewhere classified	518.89	-	2
	Primary atelectasis	770.4	-	1
	Pulmonary artery coarctation and atresia	747.31	-	1
	Respiratory bronchiolitis interstitial lung disease	516.34	-	1
	Stenosis of pulmonary artery	-	Q25.6	1
	Unspecified chronic bronchitis	491.9	-	2
Malnutrition	Malnutrition of mild degree	263.1	-	1
	Malnutrition of moderate degree	263.0	-	1
Neoplastic disease	Lymphoid leukemia acute	204.0	-	1
Neurological/neuromuscular	Cerebral palsy	-	G80	1
	Cerebral palsy, unspecified	-	G80.9	3
	Certain congenital musculoskeletal deformities	754	-	1

	Chromosomal anomalies	758	-	1
	Congenital hypotonia	-	P94.2	1
	Congenital musculoskeletal deformities of skull, face, and jaw	754.0	-	7
	Congenital reduction deformities of brain	742.2	-	1
	Congenital quadriplegia	343.2	-	2
	Down's syndrome	758.0	-	6
	Down syndrome, unspecified	-	Q90.9	3
	Hemiplegia, unspecified, affecting unspecified side	342.90	-	1
	Infantile cerebral palsy, unspecified	343.9	-	10
	Late effects of cerebrovascular disease, hemiplegia affecting unspecified side	438.20	-	1
	Microcephalus	742.1	-	4
	Other deletions of part of a chromosome	-	Q93.5	1
	Other specified paralytic syndrome	344.89	-	1
	Paralysis, unspecified	344.9	-	1
	Paraplegia (paraparesis) and quadriplegia (quadriparesis)	-	G82	1
	Unspecified congenital anomaly of brain, spinal cord, and nervous system	742.9	-	1
Premature	31-32 completed weeks of gestation	765.26	-	1
	35-36 completed weeks of gestation	765.28	-	2
	Disorders relating to other preterm infants	765.1	-	3
	Other preterm infants, 1,000-1,249 grams	765.14	-	1
	Other preterm infants, 2,500 grams and over	765.19	-	1
Renal disease	Chronic kidney disease, unspecified	-	N18.9	1
	Disorder of kidney and ureter, unspecified	-	N28.9	1
	Other pyelonephritis or pyonephrosis not specified as acute or chronic	590.8	-	2
	Renal dysplasia	753.15	Q61.4	2

Table S3. Correspondence between comorbidities identified directly from medical chart and those from ICD discharge codes combined for the analysis

Comorbidity category	Comorbidity extracted from medical chart	Comorbidity based on ICD discharge codes [a]
Anemia	Anemia	Anemia
Recurrent wheezing / asthma	Asthma	Asthma
Chronic autoimmune disease	Acquired or hereditary immunodeficiencies<br><br>Chronic autoimmune disease (lupus, rheumatoid arthritis, other)	Autoimmune
Diabetes	Diabetes	Diabetes
Endocrine system disease other than diabetes	Endocrine system disease other than diabetes (for example, hypothyroidism, hyperthyroidism)	Endocrine system disease other than diabetes
Heart disease	Heart disease	Heart disease
Chronic renal disease	Alterations of the renal function	Renal disease
Chronic liver disease	Chronic liver disease	Liver disease
Neoplastic disease	Neoplastic disease (cancer) active or under treatment (does not include cutaneous neoplasm)	Neoplastic disease

Neurological / neuromuscular diseases	Neuromuscular or neurodegenerative disease	Neurological/neuromuscular
Lung disease	Bronchitis, or COPD or any other than asthma	Lung disease
Prematurity	Gestational age at birth < 37 weeks	Premature

[a] Categories defined in Table S2

Table S4. International Classification of Diseases (ICD) codes by pathogens

Etiology	ICD code description	ICD-9	ICD-10	n
Adenovirus	Enteritis due to adenovirus	008.62	-	3
Astrovirus	Enteritis due to astrovirus	008.66	-	1
Bordetella pertussis	Whooping cough due to Bordetella pertussis	-	A37.0	1
	Whooping cough due to Bordetella pertussis [B. pertussis]	033.0	-	1
Campylobacter	Campylobacter enteritis	-	A04.5	1
	Intestinal infection due to campylobacter	008.43	-	4
Chlamydial pneumonia	Chlamydial pneumonia	-	J16, J16.0	3
Coxsackie virus	Specific diseases due to coxsackie virus	74, 074		2
Cytomegalovirus	Cytomegaloviral disease	078.5		1
	Cytomegaloviral disease, unspecified	-	B25.9	1
Enterovirus	Hand, foot, and mouth disease	074.3		2
	Meningitis due to enterovirus	47, 047		1
Epstein-Barr virus	Echo virus infection in conditions classified elsewhere and of unspecified site	079.1		1
	Infectious mononucleosis	75, 075		20
	Infectious mononucleosis, unspecified	-	B27.9	1
Escherichia coli	Escherichia coli [E. coli] as the cause of diseases classified to other chapters	-	B96.2	1
	Escherichia coli [e. coli] infection in conditions classified elsewhere and of unspecified site	041.4		19
	Other and unspecified Escherichia coli [E. coli]	041.49		2
Gammaherpesviral	Gammaherpesviral mononucleosis	-	B27.0	1
Hemophilus influenzae	Haemophilus influenzae infection, unspecified site	-	A49.2	1
	Hemophilus influenzae [H. influenzae] as the cause of diseases classified elsewhere	-	B96.3	1
	Hemophilus influenzae [H. influenzae] infection in conditions classified elsewhere and of unspecified site	041.5	-	31
	Pneumonia due to Hemophilus influenzae [H. influenzae]	482.2	-	1
Herpes simplex	Herpes simplex with other ophthalmic complications	054.49	-	1
	Herpes simplex without mention of complication	054.9	-	5
Human immunodeficiency virus	Human immunodeficiency virus [HIV] disease	042	-	3
Influenza	Influenza	487	-	26
	Influenza due to certain identified influenza viruses	488	-	10
	Influenza due to identified 2009 h1n1 influenza virus	488.1	-	8
	Influenza due to identified 2009 H1N1 influenza virus with other manifestations	488.19	-	4
	Influenza due to identified 2009 H1N1 influenza virus with other respiratory manifestations	488.12	-	23
	Influenza due to identified 2009 H1N1 influenza virus with pneumonia	488.11	-	10
	Influenza due to identified avian influenza virus	488.0	-	2
	Influenza due to identified avian influenza virus with other manifestations	488.09	-	1
	Influenza due to identified avian influenza virus with other respiratory manifestations	488.02	-	11

	Influenza due to identified avian influenza virus with pneumonia	488.01	-	2
	Influenza due to identified novel influenza A virus with other respiratory manifestations	488.82	-	5
	Influenza due to identified novel influenza A virus with pneumonia	488.81	-	5
	Influenza due to identified seasonal influenza virus	-	J10	18
	Influenza due to identified zoonotic or pandemic influenza virus	-	J09	31
	Influenza with other manifestations	487.8	-	7
	Influenza with other manifestations, seasonal influenza virus identified	-	J10.8	1
	Influenza with other respiratory manifestations	487.1	-	27
	Influenza with other respiratory manifestations, seasonal influenza virus identified	-	J10.1	9
	Influenza with other respiratory manifestations, virus not identified	-	J11.1	32
	Influenza with pneumonia	487.0	-	2
	Influenza with pneumonia, seasonal influenza virus identified	-	J10.0	4
	Influenza with pneumonia, virus not identified	-	J11.0	9
Leishmania donovani	Visceral [kala-azar] leishmaniasis	085.0	-	1
	Visceral leishmaniasis	-	B55.0	1
Measles	Measles without mention of complication	055.9	-	1
Meningococcus	Meningococcal meningitis	036.0	-	1
Metapneumovirus	Acute bronchiolitis due to human metapneumovirus	-	J21.1	2
Mycobacterium tuberculosis	Pulmonary tuberculosis, unspecified, tubercle bacilli not found (in sputum) by microscopy, but found by bacterial culture	011.94	-	1
	Pulmonary tuberculosis, unspecified, unspecified	011.90	-	1
Mycoplasma	Other specified bacterial infections in conditions classified elsewhere and of unspecified site, mycoplasma	041.81	-	1
	Pneumonia due to mycoplasma pneumoniae	483.0	J15.7	6
Parainfluenza virus	Parainfluenza virus pneumonia	-	J12.2	2
Paramyxovirus	Mumps	-	B26	1
Parvovirus B19	Erythema infectiosum (fifth disease)	057.0	-	1
Pseudomonas	Pseudomonas infection in conditions classified elsewhere and of unspecified site	041.7	-	1
Respiratory syncytial virus	Acute bronchiolitis due to respiratory syncytial virus	-	J21.0	154
	Acute bronchiolitis due to respiratory syncytial virus (RSV)	466.11	-	777
	Acute bronchitis due to respiratory syncytial virus	-	J20.5	10
	Pneumonia due to respiratory syncytial virus	480.1	-	10
	Respiratory syncytial virus (RSV)	079.6	-	109
	Respiratory syncytial virus as the cause of diseases classified elsewhere	-	B97.4	60
	Respiratory syncytial virus pneumonia	-	J12.1	21
Rhinovirus	Rhinovirus infection in conditions classified elsewhere and of unspecified site	079.3		1
Rotavirus	Enteritis due to rotavirus	008.61	-	61
	Rotaviral enteritis	-	A08.0	5
Salmonella enteritis	Salmonella enteritis	-	A02.0	3
	Salmonella gastroenteritis	003.0	-	8
Staphylococcus aureus	Methicillin resistant Staphylococcus aureus in conditions classified elsewhere and of unspecified site	041.12	-	2
	Methicillin susceptible Staphylococcus aureus in conditions classified elsewhere and of unspecified site	041.11	-	6
Streptococcus	Streptococcus infection in conditions classified elsewhere and of unspecified site	041.0	-	1
	Streptococcus infection in conditions classified elsewhere and of unspecified site, other streptococcus	041.09	-	5

	Streptococcus infection in conditions classified elsewhere and of unspecified site, streptococcus, group A	041.01	-	2
	Streptococcus infection in conditions classified elsewhere and of unspecified site, streptococcus, unspecified	041.00	-	1
	Streptococcal pharyngitis	-	J02.0	1
	Streptococcal sore throat	034.0	-	7
	Streptococcal sore throat and scarlet fever	34	-	1
Streptococcus pneumonia	Pneumonia due to Streptococcus pneumoniae	-	J13	3
	Pneumococcal meningitis	320.1	-	1
	Pneumococcal pneumonia [Streptococcus pneumoniae pneumonia]	481	-	34
	Pneumococcal septicemia [Streptococcus pneumoniae septicemia]	038.2	-	2
	Pneumococcus infection in conditions classified elsewhere and of unspecified site	041.2	-	1
Varicella	Varicella without mention of complication	052.9	-	3
Viral hepatitis	Hepatitis in viral diseases classified elsewhere	573.1	-	1
	Unspecified viral hepatitis without mention of hepatic coma	070.9	-	1
Yersinia enterocolitica	Intestinal infection due to yersinia enterocolitica	008.44	-	1

Table S5. Viral pathogens detected by RT-PCR assays by presence of comorbidity

	Total (n=6069)		Otherwise healthy (n=4469)		With at least 1 comorbidity (n=1600)	
RT-PCR results [a]	n	%	n	%	n	%
No virus pathogen detected	2790	46.0	1990	44.5	800	50.0
Influenza [b]	437	7.2	328	7.3	109	6.8
Respiratory syncytial virus	1402	23.1	1085	24.3	317	19.8
Human metapneumovirus	116	1.9	88	2.0	28	1.7
Parainfluenza	39	0.6	27	0.6	12	0.7
Rhinovirus/enterovirus	579	9.5	406	9.1	173	10.8
Adenovirus	95	1.6	80	1.8	15	0.9
Human coronavirus	197	3.2	136	3.0	61	3.8
Bocavirus	110	1.8	84	1.9	26	1.6
Codetection	281	4.6	228	5.1	53	3.3

[a] The columns don't add up to the total numbers reported in the heading of the table because of 23 underdetermined samples; p-value < 0.001.

[b] Includes Influenza A(H1N1pdm09), Influenza A(H3N2), Influenza B/Yamagata-lineage, Influenza B/Victoria-lineage and Influenza not subtyped or with no lineage.

Table S6. RT-PCR results per age group

RT-PCR results	Total (n=6069)		<6 months of age (n=2363)		6 to 11 months of age (n=677)		1 to 4 years of age (n=2339)		5 to 17 years of age (n=690)	
	n	%	n	%	n	%	n	%	n	%
No virus pathogen detected	2790	46.0	886	37.5	279	41.2	1154	49.4	471	68.3
Influenza [a]	437	7.2	100	4.2	40	5.9	213	9.1	84	12.2
Respiratory syncytial virus	1402	23.1	899	38.0	158	23.3	337	14.4	8	1.2
Human metapneumovirus	116	1.9	48	2.0	22	3.2	38	1.6	8	1.2
Parainfluenza	39	0.6	14	0.6	5	0.7	17	0.7	3	0.4
Rhinovirus/enterovirus	579	9.5	186	7.9	64	9.4	240	10.3	89	12.9
Adenovirus	95	1.6	9	0.4	10	1.5	73	3.1	3	0.4
Human coronavirus	197	3.2	78	3.3	20	2.9	81	3.5	18	2.6
Bocavirus	110	1.8	11	0.5	33	4.9	65	2.8	1	0.1
Codetection	281	4.6	123	5.2	45	6.7	110	4.7	3	0.4

[a] Includes Influenza A(H1N1pdm09), Influenza A(H3N2), Influenza B/Yamagata-lineage, Influenza B/Victoria-lineage and Influenza not subtyped or with no lineage.

Internal

Table S7. Codetections detected by RT-PCR assays

n (%)	Influenza [a]	Respiratory syncytial virus	Human metapneumovirus	Parainfluenza	Rhinovirus /enterovirus	Adenovirus	Human coronavirus	Bocavirus
2 (0.7)	x	x						
1 (0.4)	x				x			
1 (0.4)	x						x	
9 (3.2)	x	x						
1 (0.4)	x		x					
2 (0.7)	x				x			
5 (1.8)	x					x		
1 (0.4)	x						x	
1 (0.4)	x				x			
1 (0.4)	x					x		
6 (2.1)		x [b]						
3 (1.1)		x	x					
1 (0.4)		x		x				
59 (21)		x			x			
4 (1.4)		x				x		
56 (19.9)		x					x	
5 (1.8)			x		x			
2 (0.7)			x			x		
2 (0.7)			x				x	
4 (1.4)				x	x			
1 (0.4)				x			x	
10 (3.6)					x	x		
15 (5.3)					x		x	
4 (1.4)						x	x	
2 (0.7)	x							x
1 (0.4)	x	x					x	
1 (0.4)	x							x
28 (10)		x						x

1 (0.4)		x	x		x			
2 (0.7)		x			x		x	
6 (2.1)	x	x						
1 (0.4)			x		x		x	
1 (0.4)	x		x					
19 (6.8)					x			x
3 (1.1)	x				x			
5 (1.8)						x		x
9 (3.2)							x	x
1 (0.4)	x						x	
1 (0.4)	x						x	x
2 (0.7)		x			x			x
1 (0.4)		x					x	x
1 (0.4)					x	x		x

[a] Includes Influenza A(H1N1pdm09), Influenza A(H3N2), Influenza B/Yamagata-lineage, Influenza B/Victoria-lineage and Influenza not subtyped or with no lineage.
[b] coinfection of both RSV A and RSB B

Table S8. Selected complications by viral pathogens detected by RT-PCR assays

(A) Overall population

Selected complications (most commonly reported)	No virus detected (n=2790) n (%)	Influenza [a] (n=437) n (%)	Respiratory syncytial virus (n=1402) n (%)	Human metapneumo virus (n=116) n (%)	Parainfluenza (n=39) n (%)	Rhinovirus/ enterovirus (n=579) n (%)	Adenovirus (n=95) n (%)	Seasonal Human coronavirus (n=197) n (%)	Bocavirus (n=110) n (%)	Codetection (n=281) n (%)
At least 1 complication [b]	2254 (80.8)	260 (59.5)	1275 (90.9)	94 (81.0)	33 (84.6)	409 (70.6)	162 (82.2)	71 (74.7	93 (84.6)	240 (85.4)
Bronchitis/Bronchiolitis	836 (30.0)	50 (11.4)	1038 (74.0)	56 (48.3)	16 (41.0)	200 (34.5)	63 (32.0)	19 (20.0)	46 (41.8)	148 (52.7)
Pneumonia	618 (22.1)	80 (18.3)	190 (13.5)	32 (27.6)	5 (12.8)	79 (13.6)	35 (17.8)	28 (29.5)	29 (26.4)	63 (22.4)
Asthma exacerbation / Reactive airways	270 (9.7)	15 (3.4)	76 (5.4)	10 (8.6)	1 (2.6)	64 (11.0)	7 (3.5)	3 (3.2)	10 (9.1)	17 (6.1)
Febrile seizures	198 (7.1)	60 (13.7)	17 (1.2)	0 (0)	4 (10.3)	20 (3.4)	19 (9.7)	12 (12.6)	8 (7.3)	13 (4.6)
Diarrhea/Colitis	169 (6.1)	16 (3.7)	21 (1.5)	0 (0)	1 (2.6)	13 (2.2)	13 (6.6)	6 (6.3)	2 (1.8)	10 (3.6)
Other	643 (23.0)	88 (20.1)	143 (10.2)	12 (10.3)	12 (30.8)	80 (13.8)	25 (26.3)	57 (28.9)	19 (17.3)	33 (11.7)

[a] Includes Influenza A(H1N1pdm09), Influenza A(H3N2), Influenza B/Yamagata-lineage, Influenza B/Victoria-lineage and Influenza not subtyped or with no lineage.

[b] The rows below don't add up to the numbers reported in this row as a same patient may have more than one complication

(B) Otherwise healthy patients

Selected complications (most commonly reported)	No virus detected (n=1990) n (%)	Influenza [a] (n=328) n (%)	Respiratory syncytial virus (n=1085) n (%)	Human metapneumo virus (n=88) n (%)	Parainfluenza (n=27) n (%)	Rhinovirus/ enterovirus (n=406) n (%)	Adenovirus (n=80) n (%)	Seasonal Human coronavirus (n=136) n (%)	Bocavirus (n=84) n (%)	Codetection (n=228) n (%)
At least 1 complication	1595 (80.1)	191 (58.2)	980 (90.3)	75 (85.2)	22 (81.5)	288 (70.9)	60 (75.0)	109 (80.2)	74 (88.1)	15 (85.5)
Bronchitis/Bronchiolitis	629 (31.6)	36 (11.0)	811 (74.8)	43 (48.9)	10 (37.0)	149 (36.7)	13 (16.2)	40 (29.4)	37 (44.1)	118 (51.7)
Pneumonia	424 (21.3)	56 (17.1)	132 (12.2)	28 (31.8)	4 (14.8)	51 (12.6)	25 (31.2)	17 (12.5)	22 (26.2)	50 (21.9)
Asthma exacerbation / Reactive airways	137 (6.9)	6 (1.8)	46 (4.2)	9 (10.2)	0 (0)	39 (9.6)	3 (3.7)	3 (2.2)	6 (7.1)	10 (4.4)
Febrile seizures	153 (7.7)	48 (14.6)	11 (1.0)	0 (0)	3 (11.1)	16 (3.9)	11 (13.7)	17 (12.5)	8 (9.5)	11 (4.8)
Diarrhea/Colitis	131 (6.6)	14 (4.3)	15 (1.4)	0 (0)	1 (3.7)	9 (5.9)	4 (5.0)	8 (5.9)	2 (2.4)	9 (4.0)
Other	450 (22.6)	63 (19.2)	106 (9.8)	9 (10.2)	8 (29.6)	55 (13.6)	21 (26.2)	43 (31.6)	15 (17.9)	29 (12.7)

[a] Includes Influenza A(H1N1pdm09), Influenza A(H3N2), Influenza B/Yamagata-lineage, Influenza B/Victoria-lineage and Influenza not subtyped or with no lineage.

(C) Patients with at least one comorbidity

Selected complications (most commonly reported)	No virus detected (n=800) n (%)	Influenza [a] (n=109) n (%)	Respiratory syncytial virus (n=317) n (%)	Human metapneumo virus (n=28) n (%)	Parainfluenza (n=12) n (%)	Rhinovirus/ enterovirus (n=173) n (%)	Adenovirus (n=15) n (%)	Seasonal Human coronavirus (n=61) n (%)	Bocavirus (n=26) n (%)	Codetection (n=53) n (%)
At least 1 complication	660 (82.5)	69 (63.3)	295 (93.1)	19 (67.9)	11 (91.7)	121 (70.0)	11 (73.3)	53 (86.9)	19 (73.1)	45 (84.9)
Bronchitis/Bronchiolitis	207 (25.9)	14 (12.8)	227 (71.6)	13 (46.4)	6 (50.0)	51 (29.5)	6 (40)	23 (37.7)	9 (34.6)	30 (56.6)
Pneumonia	194 (24.2)	24 (22.0)	58 (18.3)	4 (14.3)	1 (8.3)	28 (16.2)	3 (20)	18 (29.5)	7 (26.9)	13 (24.5)
Asthma exacerbation / Reactive airways	133 (16.6)	9 (8.3)	30 (9.5)	1 (3.6)	1 (8.3)	25 (14.4)	0 (0)	4 (6.6)	4 (15.4)	7 (13.2)
Febrile seizures	45 (5.6)	12 (11.0)	6 (1.9)	0 (0)	1 (8.3)	4 (2.3)	1 (6.7)	2 (3.3)	0 (0)	2 (3.8)
Diarrhea/Colitis	38 (4.7)	2 (1.8)	6 (1.9)	0 (0)	0 (0)	4 (2.3)	2 (13.3)	5 (8.2)	0 (0)	1 (1.9)
Other	193 (24.1)	25 (22.9)	37 (11.7)	3 (20.8)	4 (33.3)	25 (14.4)	4 (26.7)	14 (22.9)	4 (15.4)	4 (7.5)

[a] Includes Influenza A(H1N1pdm09), Influenza A(H3N2), Influenza B/Yamagata-lineage, Influenza B/Victoria-lineage and Influenza not subtyped or with no lineage.

Table S9. Most frequent pathogens identified in ICD codes by presence of comorbidity

	Total (n=6069)		Otherwise healthy (n=4469)		With at least 1 comorbidity (n=1600)	
Viral pathogen	n	%	n	%	n	%
Respiratory syncytial virus	1093	18.0	876	19.6	217	13.6
Influenza	246	4.0	186	4.2	60	3.7
Rotavirus	66	1.1	50	1.1	16	1.0
Streptococcus pneumonia	40	0.7	25	0.6	15	0.9
Hemophilus influenzae	34	0.6	21	0.5	13	0.8
Epstein-Barr virus	22	0.4	14	0.3	8	0.5
Escherichia coli	22	0.4	18	0.4	4	0.2
Streptococcus	17	0.3	9	0.2	8	0.5
